# Growth Factors Do Not Improve Muscle Function in Young or Adult *mdx* Mice

**DOI:** 10.3390/biomedicines10020304

**Published:** 2022-01-28

**Authors:** Tue L. Nielsen, Tessa M. Hornsyld, Tomàs Pinós, Camilla Brolin, John Vissing, Thomas O. Krag

**Affiliations:** 1 Copenhagen Neuromuscular Center, Department of Neurology, Rigshospitalet, University of Copenhagen, DK-2100 Copenhagen, Denmark; tue.leth.nielsen.01@regionh.dk (T.L.N.); tmuho@dtu.dk (T.M.H.); john.vissing@regionh.dk (J.V.); 2 Mitochondrial and Neuromuscular Disorders Unit, Vall d’Hebron Institut de Recerca, Universitat Autònoma de Barcelona, 08035 Barcelona, Spain; tomas.pinos@vhir.org; 3 Centro de Investigación Biomédica en Red de Enfermedades Raras (CIBERER), 28029 Madrid, Spain; 4 Department of Cellular and Molecular Medicine, Faculty of Health and Medical Sciences, The Panum Institute, University of Copenhagen, DK-2200 Copenhagen, Denmark; cbh@sund.ku.dk

**Keywords:** *mdx*, DMD, muscular regeneration, hepatocyte growth factor, leukemia inhibitory factor

## Abstract

Muscular dystrophies constitute a broad group of genetic disorders leading to muscle wasting. We have previously demonstrated that treating a muscular atrophy mouse model with growth factors resulted in increased muscle mass. In the present study, we treated the Duchenne mouse model *mdx* for 12 weeks with myogenic growth factors peri- and post-onset of muscular degeneration to explore the effects in the oxidative muscle soleus and the glycolytic muscle extensor digitorum longus (EDL). We found no overall beneficial effect in the peri-onset group at the conclusion of the study. In the post-onset group, the functional improvement by means of electrophysiological examinations ex vivo was mostly confined to the soleus. EDL benefitted from the treatment on a molecular level but did not improve functionally. Histopathology revealed signs of inflammation at the end of treatment. In conclusion, the growth factor cocktail failed to improve the *mdx* on a functional level.

## 1. Introduction

Duchenne muscular dystrophy (OMIM, 300377: DMD) is a fatal, X-linked disease, which affects nearly 1 in 5000 newborn boys [1]. The disease is caused by absence of dystrophin, which in the healthy muscle fiber functions as a link between the sarcolemma-associated dystrophin-associated glycoprotein complex and the actin cytoskeleton apparatus of the skeletal muscle fiber, thereby connecting the contractile apparatus to the membrane [2]. Disturbances of this relationship lead to loss of sarcolemmal integrity, elevated levels of plasma creatine kinase (CK), muscular wasting, and fibro-fatty replacement of the muscle tissue [3,4]. While therapeutic strategies to rescue muscle function in DMD have been numerous, including stem cell therapy, utrophin replacement, growth factors, antisense-mediated exon-skipping, and viral reintroduction of mini/micro-dystrophins, a cure has yet to emerge.

We previously treated a hypoxia-induced muscular atrophy mouse model with growth factors (hepatocyte growth factor (HGF) and leukemia inhibitory factor (LIF)) to activate satellite cells and increase myoblast proliferation and differentiation, thus boosting what is known as the regenerative response to muscle degeneration [5]. In the muscular atrophy model, this led to a significant increase in muscle mass compared to untreated animals. This result would make the growth factor cocktail, which should be considered a potential general treatment to muscle wasting, a candidate treatment for the *mdx* mouse model of DMD, which–as patients–lacks dystrophin. Unlike other treatments that are meant to result in larger muscles, the concept of the growth factor treatment was to address both muscle wasting by increasing muscle mass and reinforce the sarcolemma, as membrane-associated proteins are upregulated as part of the maturation process of the regenerating/treated muscle fiber, by boosting the existing regenerative process. In this study, we tested if the findings from the hypoxia-induced atrophy model could be reproduced and lead to improved muscle function in the *mdx*. HGF stimulates satellite cell activation and differentiation through the cMet receptor [6,7,8] while LIF enhances myoblast survival and maintains proliferation [9,10,11]. We also introduce L-arginine, as it has been demonstrated to ameliorate the *mdx* phenotype in different distinct ways by decreasing inflammation, increasing regeneration and improving membrane integrity by upregulating utrophin [12]. Utrophin is an autosomal homolog of dystrophin which serves as a dystrophin precursor during the first 4–5 weeks of age in mice [12,13] after which it is only found in the mature muscle fiber near the neuromuscular junction [14]. However, it maintains the ability to prevent the development of muscular dystrophy [15]. In general, myofiber regeneration leads to upregulation of several membrane-associated proteins that reinforce the membrane and bind to the extra-cellular membrane.

As utrophin expression is extra-synaptic until the *mdx* is 4 to 5 weeks old, this provides an opportunity to initiate treatment peri- and post-onset of muscular degeneration, which occurs when the animals are 3–4 weeks old. Thus, to elude the possible effects of endogenous utrophin, we initiated our treatment in the *mdx* at the age of 4 and 8 weeks to determine if peri-onset treatment had a different impact from that of treating the mice post-onset. We were not able to commence treatment earlier than 4 weeks of age due to animal welfare protocols. All animals were treated for 12 weeks for the sake of comparison to previous studies [16,17,18]. We were looking for a positive effect of the treatment in ex vivo electrophysiological muscle performance-tests, since specific force production and stretch resistance are hallmarks of a translatable improvement in muscle function of the *mdx* [19]. While our approach was not designed to cure DMD by attempting to re-express dystrophin per se, we hypothesized that the treatment with growth factors would improve muscle function and that peri-onset treatment would improve muscle function more than post-onset treatment and delay or attenuate the compensatory hypertrophy, affecting specific force generation positively. This could make the combined molecular approach attractive to a larger number of myopathies with muscle wasting, where the regeneration mechanism is unaffected by the disease.

## 2. Experimental Section

### 2.1. Animals

Only male mice were used for this study. Cohorts of *mdx* (C57BL/10ScSn *Dmd^mdx^*/J) were treated and age-matched C57BL/10ScSn (wt) mice were used as the background strain, all were bred at the University of Copenhagen. Animals had access to food and water *ad libitum* and three to six mice were typically housed together in individually ventilated cages under a 12 h/12 h light/dark cycle at the animal facility of the University of Copenhagen Department of Experimental Medicine. 

### 2.2. Treatment

*Mdx* mice were randomized to receive the HGF/LIF/L-arginine growth factor cocktail (GF) or placebo (PBS). The treatment would commence when mice were either 4 (4W) or 8 weeks (8W) of age and last for 12 weeks, with the entire study consisting of six groups including wild-type mice, each with 12 male mice. Sample size of each group was based on cohort sizes previously used [16]. The treatment was given as weekly intraperitoneal injections of HGF (20 ng/g body mass, 2207HG/CF, R&D Systems, Minneapolis, MN, USA) and L-arginine (200 µg/g body mass, 11009 Sigma-Aldrich, St. Louis, MO, USA) followed by LIF (10 ng/g body mass, AMSBIO, Cambridge, MA, USA) three-to-four days later. Doses were based on previous works [5,12,13]. Body mass was recorded weekly at the time of the HGF/L-arginine injection.

### 2.3. Physiological Experiments

After receiving 12 weeks of treatment, mice were anaesthetized using Hypnorm/midazolam 0.5 mg/g body mass (Department of Experimental Medicine, University of Copenhagen, Copenhagen, Denmark) and kept on a heating pad for the dissecting procedure. Hindlimb muscles tibialis anterior (TA), extensor digitorum longus (EDL), soleus, gastrocnemius, and quadriceps were removed. The mass of all muscles was determined, and length was measured for EDL and soleus. Dissected EDL and soleus were immediately mounted on pins in the organ bath in oxygenated Krebs/Ringer solution at room temperature using 4-0 Vicryl surgical suture and left to equilibrate in oxygenated Krebs/Ringer solution at room temperature for 5 min. The electrophysiology setup consisted of an 840MD organ bath setup with force transducers and motors (DMT A/S, Aarhus, Denmark). Muscles were stimulated with 20 V·cm^−1^ using a DMT CS8 stimulator (DMT A/S). Analog signals were converted using an AD Instruments PowerLab 4/35 A/D converter and recorded using Chart 7.0 (AD Instruments, Oxford, UK). Stimulation with 5 ms square pulses for isometric twitches were used to determine the optimal base tension and base tension length, *L*_0_. After this, maximum isometric tetani were determined by stimulating the muscle with 5 ms pulses at 100 Hz for EDL and 80 Hz for soleus for 695 ms (EDL) or 705 ms (soleus) with 60 s between five trains. Muscles were then stimulated at a 50% duty cycle using 5 ms pulses for EDL and 6.5 ms pulses for soleus. Following a resting period of 5 min, the muscle was subjected to a stretch protocol, with stimulation for 700 ms of 100 Hz (EDL) or 80 Hz (soleus). After 500 ms of isometric contraction, stretch was applied to induce an eccentric contraction for the remaining 200 ms by stretching the muscle 10% of the base tension length, *L*_0_. The stretch protocol was repeated four times with a 60 s resting period in between, as previously described [16,17,20]. The maximal contraction of the five performed was used in the results. The person operating and recording muscular function of *mdx* mice was blinded to treatment. Muscles were flash-frozen in isopentane cooled by liquid nitrogen and stored at −80 °C. Physiological cross-sectional area (CSA) was determined by dividing the mass of the muscle (EDL and soleus) with the density of mammalian muscle (1.056 g·cm^−3^) and resting length, *l*_o_. Specific force was obtained by dividing the measured absolute force with the CSA. If both had been recorded, muscle mass as well as CSA for an individual animal was recorded as the average of both right and left side muscles. All results are presented as mean with standard deviations unless otherwise stated. The primary outcome of the treatment was change in specific force and stretch resistance whereas secondary outcomes such as changes in body mass, absolute force, muscle mass and CSA, and molecular markers of muscular regeneration were to support the primary findings.

### 2.4. Western Blot Analyses

TA, EDL, and soleus muscles from animals were sectioned on a cryostat and dissolved in lysis buffer (10 mM Tris, pH7.4, 0.1% Triton-X 100, 0.5% sodium deoxycholate, 0.07 U/mL aprotinin, 20 µM leupeptin, 20 µM pepstatin, 1 mM phenylmethanesulfonyl fluoride (PMSF), 1 mM ethylenediaminetetraacetic acid (EDTA), 1 mM ethylene glycol-bis(β-aminoethyl ether)-N,N,N′,N′-tetraacetic acid (EGTA), 1 mM dithiothreitol (DTT), 5 mM β-glycerophosphate, 1 mM sodium fluoride, 1.15 mM sodium molybdate, 2 mM sodium pyrophosphate decahydrate, 1 mM sodium orthovanadate, 4 mM sodium tartrate, 2 mM imidazole, 10 nM calyculin, and 5 µM cantharidin, (all Sigma-Aldrich) using a Bullet Blender tissue homogenizer at 4 °C (Next Advance Inc., Averill, NY, USA). Samples were mixed with 4× sample buffer (12.5% glycerol, 0.2 g/mL sodium dodecyl sulfate, 0.08 M bromophenol-blue, and 20% β-mercaptoethanol). Equal amounts of extracted muscle proteins were separated on 4–15% and 7.5% TGX polyacrylamide gels (Bio-Rad Laboratories Inc., Hercules, CA, USA) at 200V for 60 min (4–15% gels) and 80 min (7% gels). Proteins were blotted to polyvinylidene difluoride membranes (Bio-Rad Laboratories Inc.) at 2.5 A for 7 min using a Trans-Blot Turbo (Bio-Rad Laboratories Inc.), and 18 h at 0.6 A using a Criterion tank blotting chamber, cooled at 4 °C. Membranes were then blocked using Baileys Irish Cream (Baileys, Dublin, Ireland). After washing excess Bailey’s with TBS-T, membranes were incubated overnight in 0.5% skimmed milk with antibodies against vinculin at 1:2000 (ab73412) from Abcam (Cambridge, UK), myogenin at 1:500 (F5D) from Developmental Studies Hybridoma Bank (Iowa City, IA, USA), and myoD1 at 1:500 (clone 5.8A, MA1-41017) from Vector Laboratories (Burlingame, CA, USA). Secondary antibodies (goat anti-rabbit and goat anti-mouse) coupled with horseradish peroxidase from DAKO (Glostrup, Denmark) were diluted at 1:10,000 and used to detect primary antibodies. Immuno-reactive bands were detected using SuperSignal West Dura Extended Duration Substrate kit (Thermo Scientific, Rockford, IL, USA) and Clarity Max Western ECL substrate (Bio-Rad Laboratories Inc.). Quantification took place using a ChemiDoc™ MP Imaging system and Image Lab™-software (Bio-Rad Laboratories Inc.) was used to measure the intensities of immune-reactive bands on 16-bit digital photos. All immuno-reactive band intensities were normalized to the intensity of the vinculin bands for each subject to correct for differences in total muscle protein loaded on the gel. Subsequently, all normalized values from the group of a treatment regime were again normalized to the average of soleus or PBS in order to compare the groups and muscles, separately. Results are presented as the fold change of exercised animals from the average of soleus or PBS. All results are presented with standard deviations.

### 2.5. Histochemical Analysis

Cryo-sections of TA were stained with hematoxylin and eosin (H&E) for general histopathological evaluation. Wheat germ agglutinin (WGA) Alexa Fluor™ 647stain (ThermoFisher, Waltham, MA, USA) was diluted 1:200 in Hanks Balanced Salt Solution and applied to muscle sections to visualize fibrosis [21].

### 2.6. Immunohistochemistry

For immunohistochemistry (IHC), sections were fixed in 10% normal phosphate buffered formalin and subsequently blocked in 5% fetal bovine serum in PBS prior to staining. To assess the number of satellite cells undergoing divisions, sections were incubated with Pax7 (DSHB) diluted 1:50 and Ki67 (#15580, Abcam, Cambridge, UK) antibodies diluted 1:500. Positive nuclei were confirmed by DAPI nuclear stain (ThermoFisher). Secondary goat anti-mouse and anti-rabbit Alexa Fluor antibodies were used at a 1:500 dilution in PBS buffer (ThermoFisher). For muscle-fiber-type analysis, we used antibodies against MHC type I (MHC I) (clone BA-D5 at 1:50, DSHB), MHC IIA and IIB (clones SC-71 at 1:10 and BF-F3 at 1:50, DSHB), and MHC IIX at 1:100 (SAB2104768, Sigma-Aldrich). Secondary antibodies were Alexa Flour. At least 300 fibers per section were counted manually, by random selections of each muscle cross-section. All sections were observed at room temperature using a Nikon 10x Plan Apo or a 20× Plan Apo VC N/A 0.75 mounted on a Nikon Eclipse 80i or a Nikon Ti-E epi-fluorescence microscope (Nikon Instruments, Melville, NY, USA). Images were acquired with a 5M pixel Nikon DS-5Mc (brightfield) and 5Mpixel Andor Neo (epi-fluorescence) cameras (Andor, Belfast, Northern Ireland) using NIS-Elements Basic Research and Advanced Research (BR/AR) software and merged in software (Nikon).

### 2.7. Statistical Analysis

Two-way analysis of variance (ANOVA) was performed on data using XLSTAT (Addionsoft (2020), New York, USA. https://www.xlstat.com, accessed on 2 November 2020) with Tukey’s HSD *post*-test to show differences between variables. A *p*-value of <0.05 was considered significant.

## 3. Results

### 3.1. Body Mass

**Figure 1:**
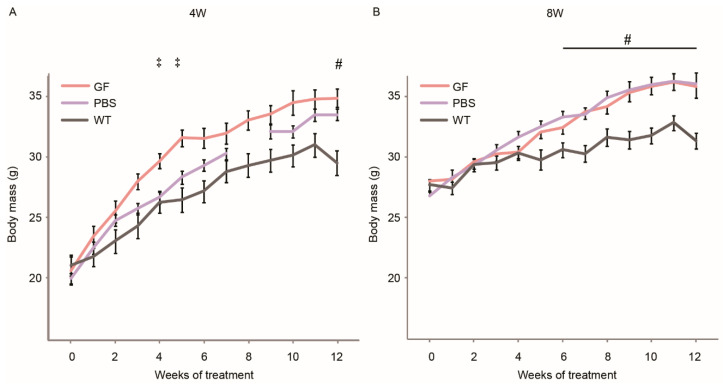
Growth curves of the different treatment cohorts. The growth pattern of the six groups in the 4W cohort (**A**) and the 8W cohort (**B**). Lack of data point in 4W PBS is due to accidental loss of measurements. GF, growth factor; PBS, phosphate-buffered saline; WT, wild-type strain. Vertical bars are SEM. Two-way ANOVAs were performed with subsequent Tukey HSD post-hoc tests to assess significance. Symbols indicate *p* < 0.05: ‡; GF vs. PBS, #; PBS vs. WT. The number N of each group was 12.

In order to assess the influence of the different treatment regimens on the growth of the mice, we followed the body mass of the mice during the 12-week period of treatment (Figure 1). In the 4W group, we observed an accelerated growth in GF-treated animals, with a significantly increased body mass compared to PBS mice in weeks 4 and 5 of treatment. During the second half of the treatment period, the two curves of GF- and PBS-mice approached each other. In the 8W group, there was no effect on body mass between GF- and PBS-treated animals. The growth curves of *mdx* mice from both age-groups flattened out at the conclusion of the treatment period. WT mice did not increase mass as rapidly as any *mdx* cohort, corresponding to compensatory muscular hypertrophy in *mdx* [22,23]. After 12 weeks of treatment, there was no significant difference in body mass between the treated and untreated cohorts of *mdx* mice.

### 3.2. Muscle Morphology

**Figure 2:**
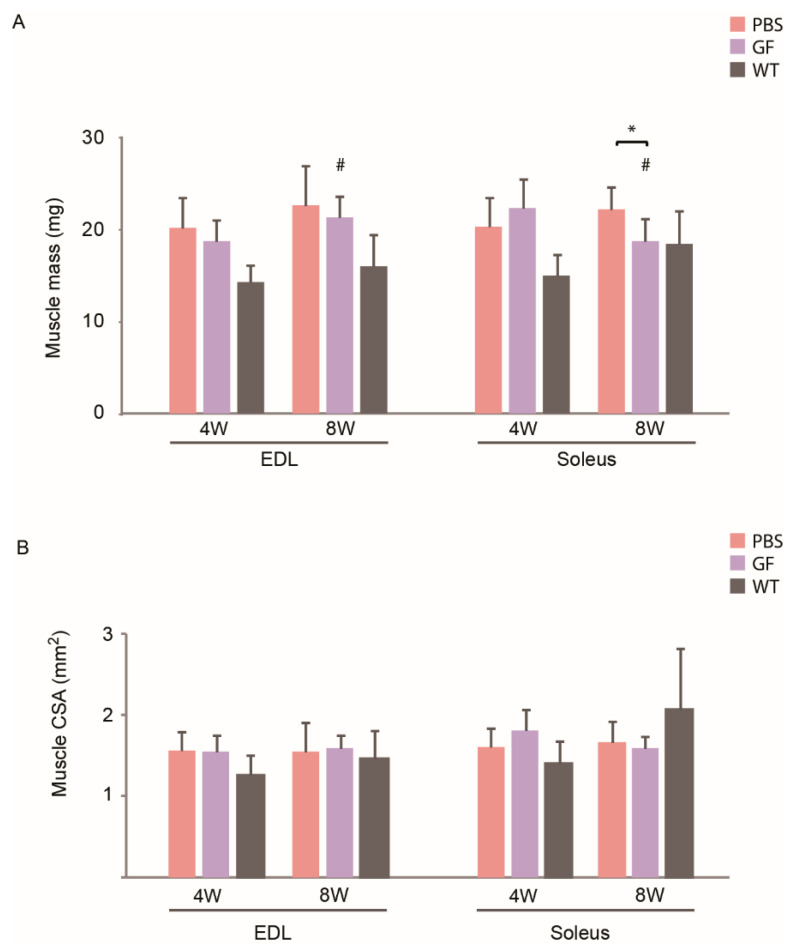
Muscle mass (**A**) and CSA (**B**) of hind-limb muscles of young (4W) and adolescent (8W) *mdx* mice treated for 12 weeks with growth factors. N for 4W animals: PBS, 12; GF, 11; WT, 11. N for 8W animals: PBS, 12; GF, 9; WT, 12. CSA, cross-sectional area; EDL, m. extensor digitorum longus; GF, growth factor; PBS, phosphate-buffered saline; WT, wild-type strain. Data presented as mean and vertical bars representing SD. Two-way ANOVAs were performed with subsequent Tukey HSD post-hoc tests to assess significance. Horizontal bars indicate significance: * *p* < 0.05, # *p* < 0.05 vs. 4W animals.

As there was no difference in body mass between treated mice and PBS controls at the conclusion of the 3-month treatment period, we were interested in observing if various muscles had responded differently to the growth-factor treatment. First, we looked at the absolute weight gain. We observed an effect of age in both EDL and soleus, as GF-treated 8W animals had a higher EDL mass (21.3 ± 5.0 mg (8W) vs. 18.7 ± 2.3 mg (4W)) but lower mass of soleus (18.7 ± 2.4 (8W) vs. 22.4 ± 3.1 (4W)) compared to 4W mice (Figure 2A). Soleus of 8W animals had a lower mass in the GF cohort compared to PBS-treated mice (18.7 ± 6.00 mg (GF) vs. 22.2 ± 6.09 mg (PBS)). There was no effect of treatment on CSA (Figure 2B). When measuring the mass of dissected hind-limb muscles m. tibialis anterior (TA), m. gastrocnemius, and m. quadriceps, no muscles in any age-group increased the mass with GF-treatment (Supplementary Materials Figure S1).

**Figure 3:**
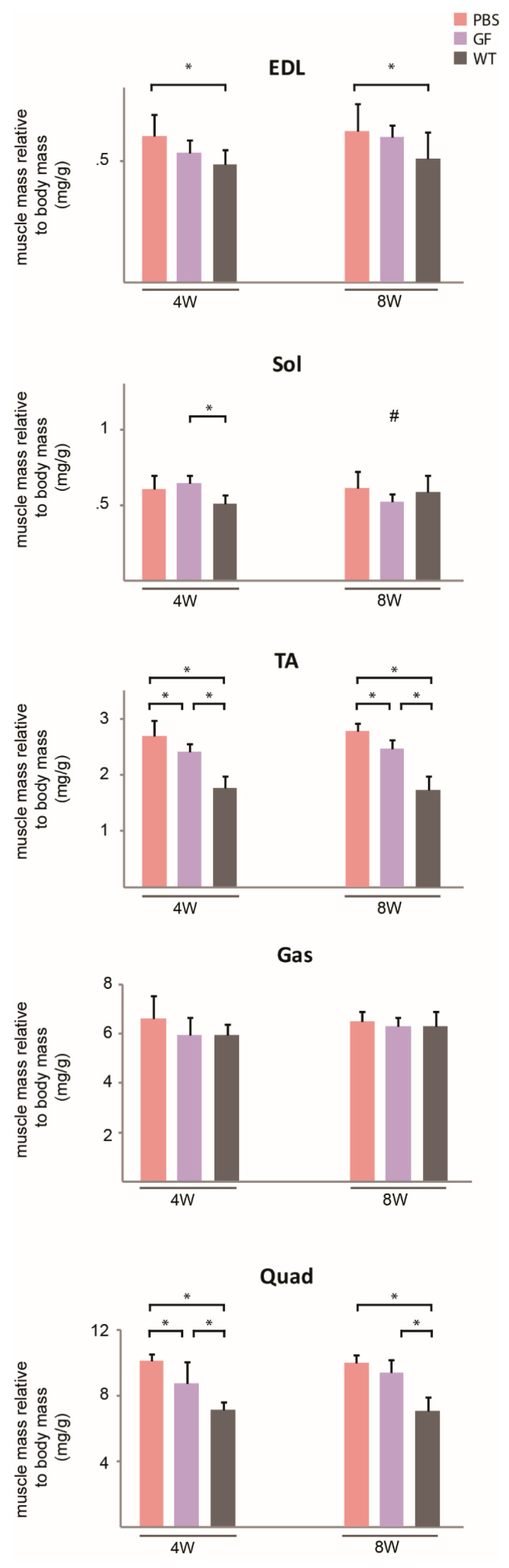
Muscle mass related to body weight. Muscle mass of EDL, soleus, TA, gastrocnemius, and quadriceps related to body mass at the conclusion of 12-week treatment period. 4W + 8W PBS; 8W WT: N = 12; 4W GF, 4W WT: N = 11; 8W GF: N = 9. EDL, m. extensor digitorum longus; Gas, m. gastrocnemius; GF, growth factor; PBS, phosphate-buffered saline; Quad, m. quadriceps; Sol, m. soleus; TA, m. tibialis anterior. Data presented as mean and vertical bars representing SD. Two-way ANOVAs were performed with subsequent Tukey HSD post-hoc tests to assess significance. Horizontal bars indicate significance: *: *p* < 0.05, #: *p* < 0.05 vs. 4W.

Second, as there was no difference between *mdx* body mass in each age group at the conclusion of treatment, we investigated how muscle mass related to body mass (Figure 3). In both age groups, EDL, TA, and quadriceps revealed that in untreated *mdx* mice, these muscles were relatively larger than in WT animals. In 4W *mdx*, TA and quadriceps treated with GF had a relatively lower mass than in untreated mice. In 8W *mdx*, only TA had a lower relative mass than PBS.

### 3.3. The Effect of Treatment on Muscle Mass across Hind-Limb Muscles and Effect on Fiber-Type Composition

**Figure 4:**
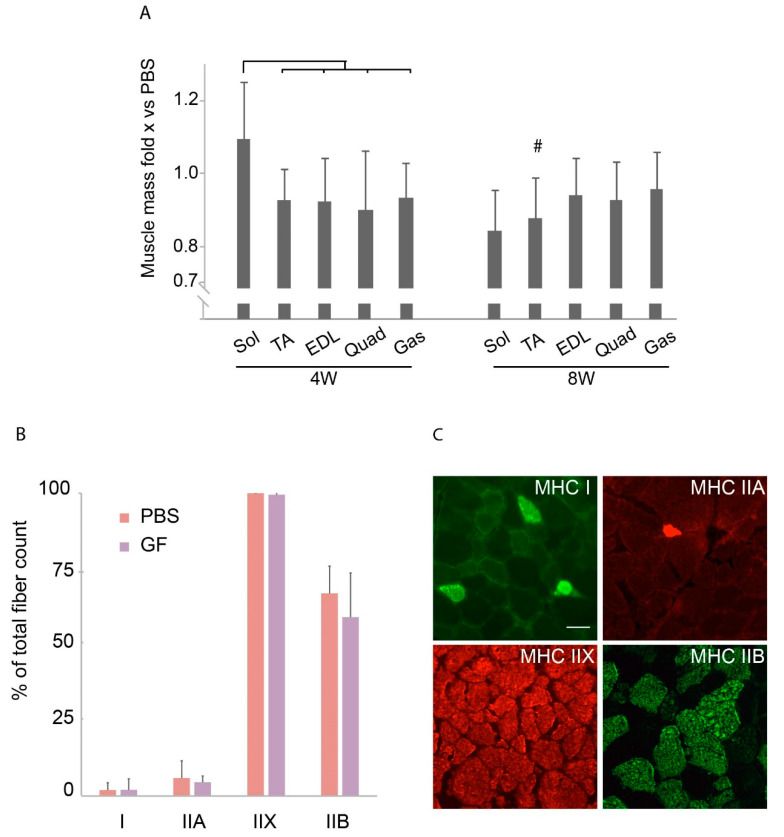
Muscle mass of treatment groups in relation to PBS and fiber-type composition. (**A**) Muscle mass of various muscles treated with growth factors compared to PBS in the two age-groups. *N* = 11 for 4W group, *N* = 9 for 8W group. (**B**) Percentage of each fiber-type present in TA in treated (*N* = 7) versus untreated (*N* = 6) 8W mice. The total distribution exceeded 100% since mixed fibers were counted twice. (**C**) Representative stains of fiber-types MHC I, IIA, IIX, and IIB. Bar represent 50 µm. EDL, m. extensor digitorum longus; Gas, m. gastrocnemius; PBS, phosphate-buffered saline; Quad, m. quadriceps; Sol, m. soleus; TA, m. tibialis anterior. Data presented as mean and vertical bars representing SD. Two-way ANOVAs were performed with subsequent Tukey HSD post-hoc tests to assess significance. Bars: *p* < 0.05, #: *p* < 0.05 vs. PBS.

To further assess a muscle-specific response to the treatment, we related the mass of dissected hind-limb muscles to that of PBS controls to tell if any muscles experienced a differentiated growth in response to treatment. This may be of interest, as we believe that muscle-fiber-type composition, which varies across the investigated muscles [24], modulates the effect of growth factors. We found, that compared to remaining muscles of the 4W group, soleus had a larger response to GF treatment than the others (Figure 4A). There was no significant variation amongst the muscles in the 8W group. Treatment did not evoke any fiber-type shift in TA which mainly consists of IIX- and IIB fibers (Figure 4B,C).

### 3.4. Physiological Studies

**Figure 5:**
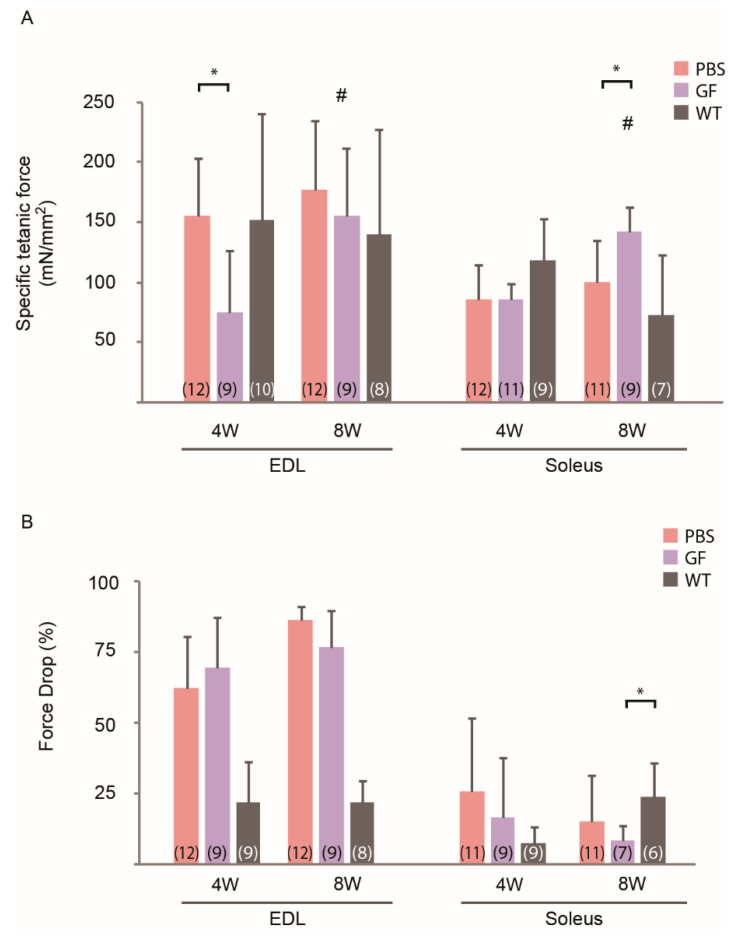
Ex vivo specific tetanic force and force drop in EDL and soleus of *mdx* treated for 12 weeks with growth factors. (**A**) Specific force of tetanic contraction in EDL and soleus of 4W and 8W *mdx*. (**B**) Force drop in muscles subjected to a stretch protocol. N is showed in the table in parentheses and may vary due to non-responsiveness to stimuli or loss of connection to the force transducer during the protocol. EDL, m. extensor digitorum longus; GF, growth factor; PBS, phosphate-buffered saline; WT, wild-type strain. Data presented as mean and vertical bars representing SD. Two-way ANOVAs were performed with subsequent Tukey HSD post-hoc tests to assess significance. Horizontal bars indicate significance: *: *p* < 0.05, #: *p* < 0.05 vs. 4W animals.

We tested EDL and soleus to determine if differences in fiber-type composition between soleus and EDL (soleus is more oxidative than EDL) would show in functional ex-vivo force measurements. An effect of age was observed in both muscles treated with GF (Figure 5A). The absolute force of EDL was surprisingly decreased by 44% (Supplementary Materials Figure S2) and specific force by 48% in GF-treated mice of the 4W group. In soleus, the treatment induced a 42% increase in specific force in 8W mice compared to PBS. We then looked at the force drop during an eccentric contraction protocol as an indicator of resistance to mechanical stress and fiber reinforcement (Figure 5B). The treatment did not significantly improve stretch resistance when comparing across treatment or age. Soleus muscles of *mdx*-mice showed better stretch resistance with an absolute force drop of 25% to 8% compared to the 62% to 86% seen in EDL.

### 3.5. Expression of Markers of Cell Division and Myogenic Signaling Proteins

**Figure 6:**
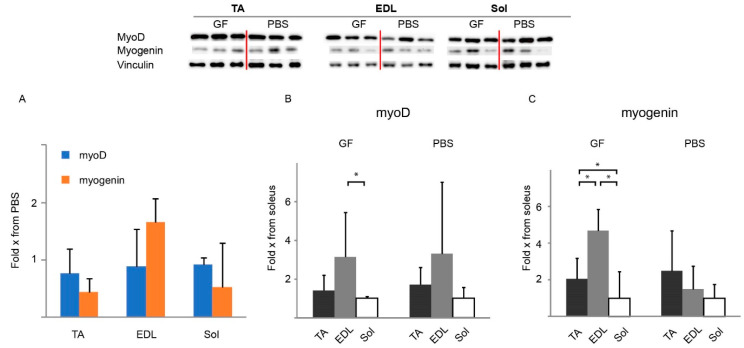
Western blotting of myogenic transcription factors in relation to PBS or soleus in adolescent (8W) *mdx* treated for 12 weeks at the conclusion of treatment. Representative bands are shown (all bands can be seen in Supplementary Materials Figure S3). (**A**) Levels of myoD and myogenin in EDL, TA, and soleus in response to GF treatment relative to PBS. (**B**,**C**) Levels of myoD (B) and myogenin (C) in various muscles relative to soleus. A: *N* = 6 for all groups. B and C: *N* = 6 for all groups except EDL of PBS group (*n* = 5). EDL, m. extensor digitorum longus; GF, growth factor; PBS, phosphate-buffered saline; Sol, m. soleus; TA, m. tibialis anterior. Data presented as bars with the respective mean and SD as vertical bar. Two-way ANOVAs were performed with subsequent Tukey HSD post-hoc tests to assess significance. Horizontal bars indicate significance: *: *p* < 0.05.

As the GF treatments either depend on or regulate the myogenic program, the level of myoD and myogenin protein expression was determined in TA, EDL, and soleus of the 8W cohort by Western blotting. MyoD and myogenin were used to assess the level of regeneration on a molecular level [25], as these markers are upregulated during stimulation with growth factors activating satellite cells [5]. Treating 8W *mdx* with GF did not yield any significant effect on either myoD or myogenin compared to PBS (Figure 6A). We then compared the expression levels across the different muscles, normalized to soleus, to examine how each muscle reacted to each treatment. Soleus was chosen as baseline since it is an oxidative muscle and has a fiber type composition that most resembles that of humans (58% type I-fibers in the *mdx* [26] and 30% in wild-type mice [24]). MyoD was significantly increased in EDL from the GF group when normalized to soleus (Figure 6B). GF treatment increased myogenin in not only EDL but also in TA compared to soleus (Figure 6C).

### 3.6. Histochemical Analysis of General Histology, Fibrosis, and Satellite-Cell Activation

**Figure 7:**
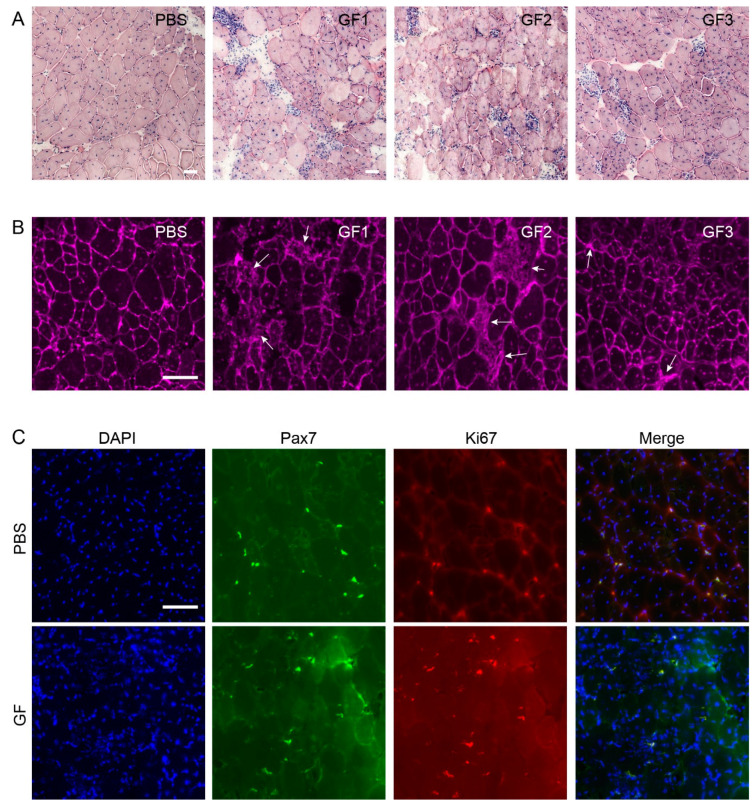
Effect of treatment on histopathology. H&E, WGA, and IHC stains of treated (GF) versus controls (PBS) *mdx* animals. (**A**) H&E stains demonstrate fiber size variation, focal inflammation, and necrotic fibers in treated animals compared to controls. (**B**) WGA showed increased fibrosis (arrows) in treated animals. (**C**) DAPI (blue), Pax7 (green), and Ki67 (red) showed activation of satellite cells. Bars represent 50 mm.

To assess the effect of treatment on histopathology in the 8W muscles, we made a qualitative analysis of H&E-stained muscle sections for general histology, and WGA stain for assessment of fibrosis. We saw extensive focal cellular infiltration and inflammation and fiber necrosis (Figure 7A) and increased fibrosis (Figure 7B) in the TA of GF-treated *mdx* animals. In addition, we found increased amounts of activated satellite cells as visualized by co-expression of Pax7 and Ki67 (Figure 7C).

## 4. Discussion

We have previously found that administration of myogenic factors to a mouse model of muscular atrophy led to a gain of muscle mass. The aim of this study was to test the hypothesis that administration of the same myogenic growth factors would ameliorate the dystrophic phenotype of the *mdx* mouse and, if so, whether there would be a difference in outcome between peri- and post-onset treatment of disease. The important findings of this study are that the greatest effect on body mass in the 4W group was seen from 4 to 5 weeks into the treatment period and that soleus of this animal group showed the greatest relative increase in mass of any muscle. Treatment induced decreased force production in EDL of young animals while the 8W group show improved force production in EDL and soleus compared to 4W mice. Changes on the molecular level in 8W animals did not translate into enhanced stress resistance, most likely due to a proinflammatory response evoked by the treatment. We found no overall advantage of peri-onset treatment compared to post-onset treatment with the growth-factor cocktail.

This is the first published study with a combination of myotrophic compounds HGF, LIF, and L-arginine specifically targeting regeneration and satellite cell proliferation in vivo in the *mdx* mouse. The effect of the treatment was immediately visualized in the development of body mass, as the 4W group treated with GF experienced accelerated growth during weeks 4 to 5 of treatment, while both curves were approaching each other at the conclusion of the study (Figure 1A). We suspected that the differences in body mass–which were absent in the 8W growth curve–would indicate that initiation of treatment around the debut of the degenerative cycles is crucial and that positive effects of this relationship would come to show in the following tests and examinations. Unfortunately, we did not find coherent improvements with regards to muscle mass, CSA or ex vivo muscle function in the 4W group.

Both HGF and LIF have been shown to regulate inflammation, and in the case if LIF both pro- and anti-inflammatory effects have been observed [27,28], which may be a relevant property to investigate if the growth factor cocktail were to be used to treat animal models less affected than the *mdx* model [29,30]. However, our study shows that either ongoing regeneration, as in the mdx, or prolonged treatment for 12 weeks tilts the effect towards proinflammation as visualized histologically (Figure 7A,B). This was an unexpected finding considering the beneficial effects in atrophy mice where the treatment was the same but shorter. Other, milder mouse models such as the novel L276I limb-girdle muscular dystrophy 2I mouse [31] could be of interest in a similar experimental setup including evaluation of inflammation.

In the 8W group, growth curves were basically identical during the 12-week treatment period and flattened out in the end of the treatment period (Figure 1B). This indicates that the regenerative and myogenic processes in developing *mdx* mice post-onset of disease, are already working at maximum level. We suspect that muscular regeneration versus degeneration has reached steady state and that any additional effect is under the control of negative regulators of muscle mass, such as myostatin [5] as demonstrated in previous works [32] or inhibited by the molecular response of inflammation. This is also in direct contrast to the similar but beneficial GF treatment of a mouse model of muscular atrophy, where the potential to increase the regenerative capacity was substantially larger since there was no ongoing degeneration–regeneration cycle [5].

In general, *mdx* mice have increased absolute force but lower specific force compared to wild-type animals [16,33]. The biggest beneficial effect of treatment was surprisingly seen in the functional studies of soleus of 8W animals (Figure 5A) compared to what was expected from the morphometrical analyses. The higher specific-force generation in soleus of GF-treated 8W-animals compared to PBS with no apparent change in CSA, seems counterintuitive, unless the sarcolemma was reinforced in the treated animals (Figure 2B). *Mdx* muscles compensate for the dystrophic properties of the myofiber by undergoing hypertrophy, explaining the greater body- and muscle mass of *mdx* compared to wild-type mice [22,23].

Western blotting was used to investigate if the functional improvement in soleus corresponded to changes in expression of myogenic factors in the 8W cohort, which is why this was done solely for this cohort. Growth factors had a positive effect on pro-myogenic transcription factors at the molecular level (Figure 6B,C), which we have previously shown in a similar study treating a muscular-atrophy model [5]. That study also demonstrated that treatment increased the ratio of Ki67- to pax7-positive nuclei and thereby the number of activated/dividing satellite cells, which we also observe in this study (Figure 7C). However, in the present study this, unfortunately, did not translate into mechanical stress resistance in EDL of 8W animals (Figure 5B) and the greater force production in soleus did not extend down to increased myoD and myogenin. This indicates that fiber reinforcement on the molecular level was insufficient to translate into amelioration of the disconnected link between the dystrophin-associated glycoprotein complex and actin, as previously noted [34,35,36] or that fiber function was hampered by inflammation. Improved resistance to mechanical stress in *mdx* animals has only been demonstrated in very few previous works [16,37,38]. The minor force-drop in soleus compared to EDL (Figure 5B) most likely reflects a combination of lower force generation, thus less stretch on the single fiber, and better endurance considering its oxidative-fiber-type composition.

The growth-factor treatment did not shift the fiber-type distribution, which was not surprising considering the overall negative findings of the study. Had there been an effect of the treatment on the MHC fiber-type composition in the *mdx*, this would most likely have shifted the isotype pattern towards those previously described in healthy mice [24], as compensatory changes would be resolved. The TA was chosen, as this muscle is composed almost entirely of IIX fibers, allowing changes towards either a more oxidative (IIA) or a more glycolytic (IIB) phenotype.

To conclude, this study failed to show overall consistent improvements in the *mdx* and did not demonstrate a coherent difference in commencing treatment peri- versus post-onset of disease. However, we have shown a potential for accelerating growth and muscular regeneration early in the disease process of the *mdx* and demonstrate a differentiated response in EDL versus soleus but also a potentially toxic effect of the treatment regime. The findings in this study add to the application of growth factors in future studies of myopathies, as additional acceleration of regeneration by a combination of myostatin inhibitors and growth factors should be examined further.

## Data Availability

The data presented in this study are available on request from the corresponding author.

## Supplementary Materials

The following supporting information can be downloaded at: https://www.mdpi.com/article/10.3390/biomedicines10020304/s1.

## References

[1] Gatheridge M.A., Kwon J.M., Mendell J.M., Scheuerbrandt G., Moat S.J., Eyskens F., Rockman-Greenberg C., Drousiotou A., Griggs R.C. (2016). Identifying Non-Duchenne Muscular Dystrophy-Positive and False Negative Results in Prior Duchenne Muscular Dystrophy Newborn Screening Programs: A Review. JAMA Neurol..

[2] Krag T.O.B., Gyrd-Hansen M., Khurana T.S. (2001). Harnessing the Potential of Dystrophin-Related Proteins for Ameliorating Duchenne’s Muscular Dystrophy. Acta Physiol. Scand..

[3] Emery A.E. (2002). The Muscular Dystrophies. Lancet.

[4] Stedman H.H., Sweeney H.L., Shrager J.B., Maguire H.C., Panettieri R.A., Petrof B., Narusawa M., Leferovich J.M., Sladky J.T., Kelly A.M. (1991). The Mdx Mouse Diaphragm Reproduces the Degenerative Changes of Duchenne Muscular Dystrophy. Nature.

[5] Hauerslev S., Vissing J., Krag T.O. (2014). Muscle Atrophy Reversed by Growth Factor Activation of Satellite Cells in a Mouse Muscle Atrophy Model. PLoS ONE.

[6] Miller K.J., Thaloor D., Matteson S., Pavlath G.K. (2000). Hepatocyte Growth Factor Affects Satellite Cell Activation and Differentiation in Regenerating Skeletal Muscle. Am. J. Physiol. Cell Physiol..

[7] Sheehan S.M., Allen R.E. (1999). Skeletal Muscle Satellite Cell Proliferation in Response to Members of the Fibroblast Growth Factor Family and Hepatocyte Growth Factor. J. Cell. Physiol..

[8] Gill R., Hitchins L., Fletcher F., Dhoot G.K. (2010). Sulf1A and HGF Regulate Satellite-Cell Growth. J. Cell Sci..

[9] White J.D., Davies M., Grounds M.D. (2001). Leukaemia Inhibitory Factor Increases Myoblast Replication and Survival and Affects Extracellular Matrix Production: Combined in Vivo and in Vitro Studies in Post-Natal Skeletal Muscle. Cell Tissue Res..

[10] Barnard W., Bower J., Brown M.A., Murphy M., Austin L. (1994). Leukemia Inhibitory Factor (LIF) Infusion Stimulates Skeletal Muscle Regeneration after Injury: Injured Muscle Expresses Lif MRNA. J. Neurol. Sci..

[11] Austin L., Bower J.J., Bennett T.M., Lynch G.S., Kapsa R., White J.D., Barnard W., Gregorevic P., Byrne E. (2000). Leukemia Inhibitory Factor Ameliorates Muscle Fiber Degeneration in the Mdx Mouse. Muscle Nerve.

[12] Chaubourt E., Fossier P., Baux G., Leprince C., Israël M., De La Porte S. (1999). Nitric Oxide and L-Arginine Cause an Accumulation of Utrophin at the Sarcolemma: A Possible Compensation for Dystrophin Loss in Duchenne Muscular Dystrophy. Neurobiol. Dis..

[13] Barton E.R., Morris L., Kawana M., Bish L.T., Toursel T. (2005). Systemic Administration of L-Arginine Benefits Mdx Skeletal Muscle Function. Muscle Nerve.

[14] Khurana T.S., Watkins S.C., Chafey P., Chelly J., Tomé F.M.S., Fardeau M., Kaplan J.-C., Kunkel L.M. (1991). Immunolocalization and Developmental Expression of Dystrophin Related Protein in Skeletal Muscle. Neuromuscul. Disord..

[15] Tinsley J., Deconinck N., Fisher R., Kahn D., Phelps S., Gillis J.M., Davies K. (1998). Expression of Full-Length Utrophin Prevents Muscular Dystrophy in Mdx Mice. Nat. Med..

[16] Bogdanovich S., Krag T.O.B., Barton E.R., Morris L.D., Whittemore L.-A., Ahima R.S., Khurana T.S. (2002). Functional Improvement of Dystrophic Muscle by Myostatin Blockade. Nature.

[17] Bogdanovich S., Perkins K.J., Krag T.O.B., Whittemore L.-A., Khurana T.S. (2005). Myostatin Propeptide-Mediated Amelioration of Dystrophic Pathophysiology. FASEB J..

[18] Pistilli E.E., Bogdanovich S., Goncalves M.D., Ahima R.S., Lachey J., Seehra J., Khurana T. (2011). Targeting the Activin Type IIB Receptor to Improve Muscle Mass and Function in the Mdx Mouse Model of Duchenne Muscular Dystrophy. Am. J. Pathol..

[19] Dellorusso C., Crawford R.W., Chamberlain J.S., Brooks S.V. (2001). Tibialis Anterior Muscles in Mdx Mice Are Highly Susceptible to Contraction-Induced Injury. J. Muscle Res. Cell Motil..

[20] Bogdanovich S., McNally E.M., Khurana T.S. (2008). Myostatin Blockade Improves Function but Not Histopathology in a Murine Model of Limb-Girdle Muscular Dystrophy 2C. Muscle Nerve.

[21] Emde B., Heinen A., Gödecke A., Bottermann K. (2014). Wheat Germ Agglutinin Staining as a Suitable Method for Detection and Quantification of Fibrosis in Cardiac Tissue after Myocardial Infarction. Eur. J. Histochem. EJH.

[22] Sacco P., Jones D.A., Dick J.R.T., Vrbová G. (1992). Contractile Properties and Susceptibility to Exercise-Induced Damage of Normal and Mdx Mouse Tibialis Anterior Muscle. Clin. Sci..

[23] Pastoret C., Sebille A. (1995). Mdx Mice Show Progressive Weakness and Muscle Deterioration with Age. J. Neurol. Sci..

[24] Bloemberg D., Quadrilatero J. (2012). Rapid Determination of Myosin Heavy Chain Expression in Rat, Mouse, and Human Skeletal Muscle Using Multicolor Immunofluorescence Analysis. PLoS ONE.

[25] Cornelison D.D.W., Wold B.J. (1997). Single-Cell Analysis of Regulatory Gene Expression in Quiescent and Activated Mouse Skeletal Muscle Satellite Cells. Dev. Biol..

[26] Carnwath J.W., Shotton D.M. (1987). Muscular Dystrophy in the Mdx Mouse: Histopathology of the Soleus and Extensor Digitorum Longus Muscles. J. Neurol. Sci..

[27] Kerr B.J., Patterson P.H. (2004). Potent Pro-Inflammatory Actions of Leukemia Inhibitory Factor in the Spinal Cord of the Adult Mouse. Exp. Neurol..

[28] McKenzie R.C., Paglia D., Kondo S., Sauder D.N. (1996). A Novel Endogenous Mediator of Cutaneous Inflammation: Leukemia Inhibitory Factor. Acta Derm. Venereol..

[29] Hunt L.C., Upadhyay A., Jazayeri J.A., Tudor E.M., White J.D. (2013). An Anti-Inflammatory Role for Leukemia Inhibitory Factor Receptor Signaling in Regenerating Skeletal Muscle. Histochem. Cell Biol..

[30] Molnarfi N., Benkhoucha M., Funakoshi H., Nakamura T., Lalive P.H. (2015). Hepatocyte Growth Factor: A Regulator of Inflammation and Autoimmunity. Autoimmun. Rev..

[31] Krag T.O., Vissing J. (2015). A New Mouse Model of Limb-Girdle Muscular Dystrophy Type 2I Homozygous for the Common L276I Mutation Mimicking the Mild Phenotype in Humans. J. Neuropathol. Exp. Neurol..

[32] Hennebry A., Oldham J., Shavlakadze T., Grounds M.D., Sheard P., Fiorotto M.L., Falconer S., Smith H.K., Berry C., Jeanplong F. (2017). IGF1 Stimulates Greater Muscle Hypertrophy in the Absence of Myostatin in Male Mice. J. Endocrinol..

[33] Barton E.R., Lynch G., Khurana T.S. (2008). Measuring Isometric Force of Isolated Mouse Muscles in Vitro. Exp. Protoc. DMD Anim. Models Treat-NMD Neuromuscul. Netw..

[34] Dumonceaux J., Marie S., Beley C., Trollet C., Vignaud A., Ferry A., Butler-Browne G., Garcia L. (2010). Combination of Myostatin Pathway Interference and Dystrophin Rescue Enhances Tetanic and Specific Force in Dystrophic Mdx Mice. Mol. Ther..

[35] Hoogaars W.M.H., Mouisel E., Pasternack A., Hulmi J.J., Relizani K., Schuelke M., Schirwis E., Garcia L., Ritvos O., Ferry A. (2012). Combined Effect of AAV-U7-Induced Dystrophin Exon Skipping and Soluble Activin Type IIB Receptor in Mdx Mice. Hum. Gene Ther..

[36] Petrof B.J., Shrager J.B., Stedman H.H., Kelly A.M., Sweeney H.L. (1993). Dystrophin Protects the Sarcolemma from Stresses Developed during Muscle Contraction. Proc. Natl. Acad. Sci. USA.

[37] Krag T.O.B., Bogdanovich S., Jensen C.J., Fischer M.D., Hansen-Schwartz J., Javazon E.H., Flake A.W., Edvinsson L., Khurana T.S. (2004). Heregulin Ameliorates the Dystrophic Phenotype in Mdx Mice. Proc. Natl. Acad. Sci. USA.

[38] Murphy K.T., Ryall J.G., Snell S.M., Nair L., Koopman R., Krasney P.A., Ibebunjo C., Holden K.S., Loria P.M., Salatto C.T. (2010). Antibody-Directed Myostatin Inhibition Improves Diaphragm Pathology in Young but Not Adult Dystrophic Mdx Mice. Am. J. Pathol..

