# Supplementary material for: Growth Factors Do Not Improve Muscle Function in Young or Adult mdx Mice
Source: Biomedicines. 2022 Jan 28;10(2):304. doi: 10.3390/biomedicines10020304 (PMC8869250; doi:10.3390/biomedicines10020304)
Supplement: Supplementary file 1 [file biomedicines-10-00304-s001.zip › biomedicines-1567379-supplementary.pdf]

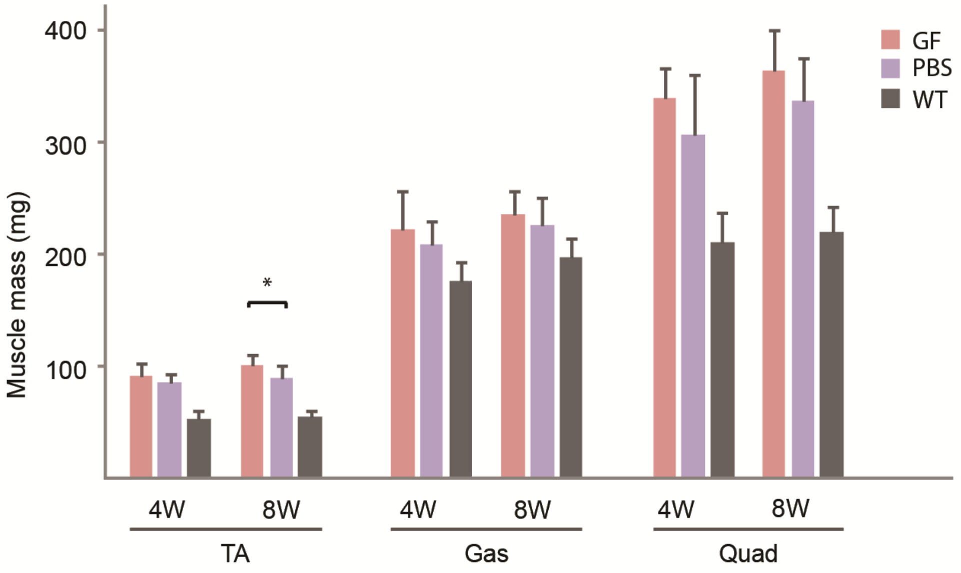

Figure S1. Muscle mass of tibialis anterior (TA), gastrocnemius (Gas) and quadriceps (Quad) of *mdx* mice treated with placebo (PBS) or growth factors (GF). N for 4W animals: PBS: 12, GF: 11, WT: 11. N for 8W animals: PBS: 12, GF: 9, WT: 12. 4W: 4-week cohort, 8W: 8-week cohort, WT: Wild type controls. Two-way ANOVAs were performed with subsequent Tukey HSD post-hoc tests to assess significance. Horizontal bars indicate significance: * $P < 0.05$, # $P < 0.05$ vs 4W animals.

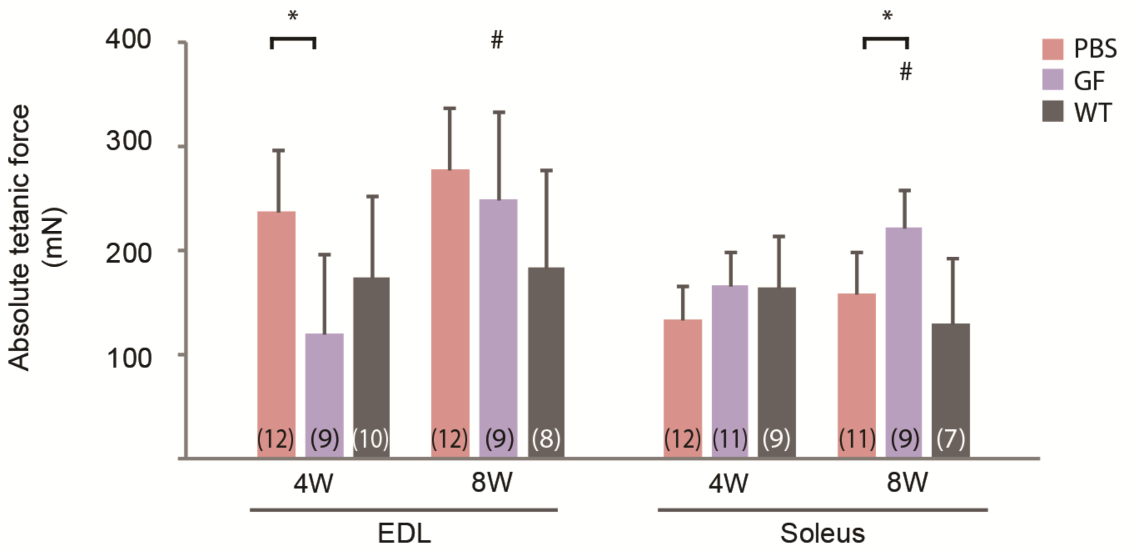

Figure S2. Ex vivo absolute tetanic force in EDL and soleus of mdx treated for 12 weeks with growth factors. N is shown in parentheses. EDL; m. extensor digitorum longus, GF; growth factor, PBS; phosphate-buffered saline, WT; wild type strain. Two-way ANOVAs were performed with subsequent Tukey HSD post-hoc tests to assess significance. Horizontal bars indicate significance: * $P < 0.05$, # $P < 0.05$ vs 4W animals.

Original blots for figure 6

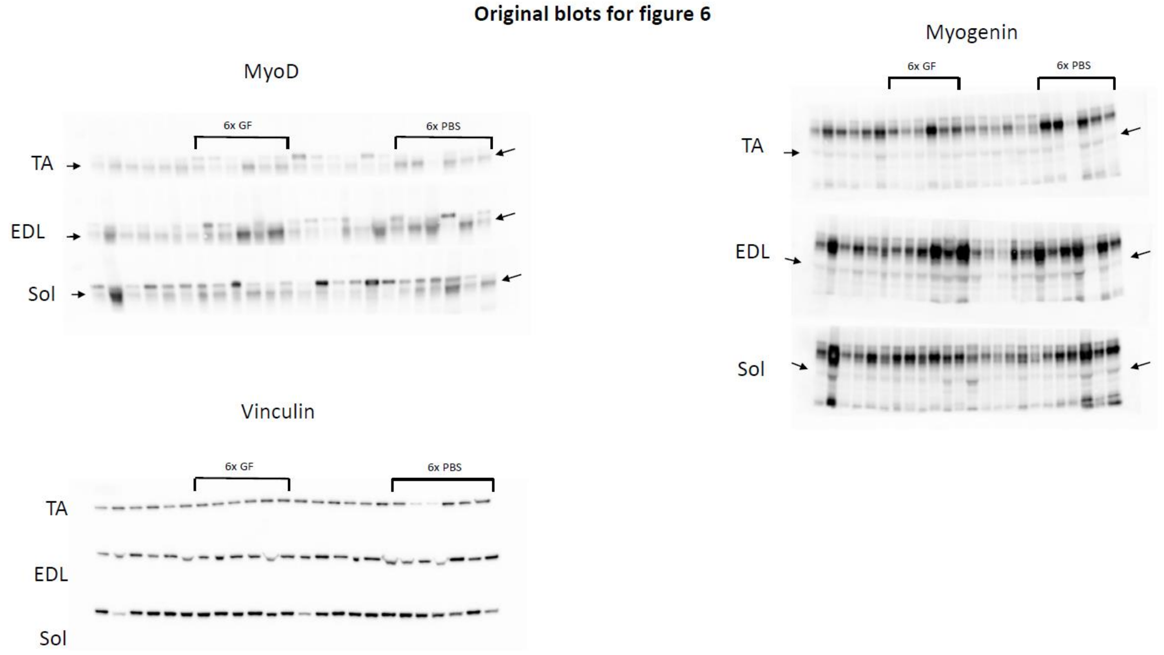

Figure S3. Original blots for Figure 6.
